# Real-world data about emotional stress, disability and need for social care in a German IBD patient cohort

**DOI:** 10.1371/journal.pone.0227309

**Published:** 2020-01-03

**Authors:** Alica Kubesch, Patric Boulahrout, Natalie Filmann, Irina Blumenstein, Johannes Hausmann

**Affiliations:** 1 Department of Internal Medicine 1, Goethe-University Hospital, Frankfurt, Germany; 2 Department 4 Social Work and Health, Frankfurt University of Applied Sciences, Frankfurt, Germany; 3 Institute of Biostatistics and Math Modeling, Goethe-University Hospital, Frankfurt, Germany; Laikon Hospital, GREECE

## Abstract

To date, there is insufficient insight into inflammatory bowel disease (IBD)-associated stress, recognized disability, and contact with the social care system. We aimed to assess these parameters in IBD patients and a non-IBD control group, who were invited to participate in an online survey developed specifically for this study (www.soscisurvey.de) with the help of IBD patients. 505 IBD patients and 166 volunteers (i.e., control group) participated in the survey. IBD patients reported significantly increased levels of stress within the last six months and five years (p<0.0001) and were more likely to have a recognized disability (p<0.0001). A low academic status was the strongest indicator of a disability (p = 0.006). Only 153 IBD patients (30.3%) reported contact with the social care system, and a disability was the strongest indicator for this (p<0.0001). Our study provides data on stress and disability in a large unselected German IBD cohort. We showed that patients with IBD suffer more often from emotional stress and more often have a recognized disability. As only about 1/3 of the patients had come into contact with the social care system and the corresponding support, this patient group is undersupplied in this area.

## Introduction

Inflammatory bowel disease (IBD) is a group of chronic and recurring inflammatory conditions of the intestine of unknown cause, which encompasses Crohn’s disease (CD) and ulcerative colitis (UC) and often affects young people. Because these diseases are intractable and require long-term therapy, patients not only undergo physical strains but also deteriorated quality of life (QoL), both mentally and socially[1]^,^[2]. Despite recent therapeutic advances, patients continue to suffer significant emotional distress.

Thus, studying and consecutively improving the patient’s QoL and determining risk factors for disability have become a great interest. However, to date, potential risk factors other than the presence of IBD itself for developing a disability and having a decreased QoL in IBD patients are still scarcely defined, and the importance of factors such as educational status is discussed controversial[3]. There is evidence that patients with IBD have lower academic status than healthy controls[4]^,^[5]. Studies on school functioning have shown that children with IBD tend to have poorer school attendance[6]–especially when having a high disease activity[7] and also appear to participate less in extra-curricular activities[8]. Therefore, improving these aspects of life, especially in young IBD patients, should be taken into consideration for holistic care. Another relevant factor that influences the QoL of IBD patients is disease-related discrimination and stigmatization[9]. Reports on grades of disability vary from 1.3 to 50% and are dependent on the definition of disability and the way the data was obtained, often through studies in highly selected patient cohorts. Real-world data (i.e., unselected patient cohorts) on this topic is lacking[10]^,^[11]. In Germany, a recognized disability is defined as having a grade of disability granted by a governmental institution. The patient’s recognized diagnoses and the resulting complaints/symptoms are taken into consideration, and the granted percentages range from 0 to 100%. Over the last decade, several valuable IBD and QoL questionnaires have been developed. However, most focus on hospital-based clinical and treatment aspects. Although there is extensive data on emotional stress, disability, anxiety, and depression in IBD patients[12]^,^ [13]^,^[14], there is only limited data derived through questionnaires constructed with the aid of IBD patients. This study aimed to provide insights into emotional stress in an unselected IBD cohort. IBD patients strongly influenced the conceptualization of this questionnaire. They suggested questions they were rarely asked but deemed necessary. Furthermore, we sought to determine if patients with IBD are more likely to have a recognized disability and whether predictive parameters associated with a recognized disability could be determined. Finally, we assessed the level of contact with the social care system. As this survey is based on a self-constructed and not validated questionnaire, it has to be considered as a pilot study.

## Material and methods

### Survey population

IBD patients and non-IBD subjects were invited to partake in an online survey between November 15^th^ and December 21^st^, 2018. This survey was accessible via www.soscisurvey.de and was approved by the German Association for Crohn’s disease and Ulcerative Colitis (DCCV). This survey was advertised through social media, the DCCV website, and associated media. Participation in this survey was voluntary, and no participant-specific data (i.e., data that would lead to the participant’s identity) were collected. Therefore no approval from the local ethics committee was needed for this study.

### Survey design

The questionnaire was created for this study by the department for social work and health, Frankfurt University of Applied Sciences, Frankfurt am Main, Germany, in cooperation with IBD patients. They were asked which aspects of life caused them emotional stress and which questions they believed they rarely got asked in standard surveys. These questions and topics were included in the conceptualization of the questionnaire. The questions focused on overall emotional stress and to determine possibly related factors. One section enquired about IBD-related disability and contact with social systems (i.e., social workers, governmental support). The questionnaire consists of 80 questions, divided into five sections. Section one focused on general data, section two on stress in daily life (past 6 months and 5 years), section three on stress at work (past 6 months and 5 years), section four on discrimination in private life and at work and section five on social work. The participants were asked to answer the question with “yes” or “no” and/or rate the respective topic on a five-point Likert scale. The International Classification of Functioning, Disability and Health (ICF) served as an orientation for the questionnaire[15]. For the entire questionnaire, please refer to the supplemental data (S1 File).

### Social care system and recognized disability

The social care system was defined as contact with and provided support by social workers, self-help groups, and governmental support. The support entailed providing information concerning IBD, self-help groups, physicians, opportunities for rehabilitation, home care, and guidance for obtaining a recognized disability. A recognized disability was defined as having a disability based on an underlying disease granted by the state after a specific application and evaluation.

**Stress in daily life and work:** This consisted of 28 questions concerning stress in everyday life and 14 questions concerning stress at work. Participants were instructed to relate their answers to the questions to either the past six months or five years. The items were identical in both subdivisions for stress in daily life or at work.

**Discrimination in daily life (i.e., private life) and work:** This section consisted of five questions concerning discrimination in everyday life and seven questions concerning discrimination at work.

**Contact with social work and evaluation:** This section of the questionnaire entailed 12 questions concerning contact and satisfaction with the social care system in Germany.

### Statistical analyses

Nominal data were presented as raw numbers and percentages, ordinal data as median and IQR, and quantitative data as mean and sd or, if skewed, as median and IQR. Quantitative data were assessed using the Wilcoxon-Mann-Whitney-U-tests. P values <0.05 were considered to be statistically significant. Associations of recognized disability with continuous or dichotomic variables were assessed in linear and logistic regression models, respectively. After univariate analyses, multivariate analyses were performed for significant associations. Multivariate models were obtained by backward selection, using a p-value >0.15 for removal from the model. Statistical analyses were conducted using IBM SPSS Statistics Version 22.0 (International Business Machine Corporation, Endicott, NY, USA).

## Results

**Patient characteristics:** 505 patients with IBD and 166 control group volunteers participated in this study.

In the IBD group, 444 (87.3%) were female. The high percentage of female participants was also observed in the UC (n = 32; 84.6%) and CD subgroups (n = 289; 89). In the control group, only 109 (65.7%) participants were female. The median age of the IBD cohort was 35 years [range 15–70], whereas the median age in the control group was 32 years [range 18–69]. In the IBD subgroup, the median age was 34.5 [range 15–63] and 35.5 years [range 16–67] for UC and CD, respectively. Participants in the control group were significantly younger than patients in the IBD group (p = 0.004). The median age was similar in both IBD subgroups (p = 0.243). Interestingly participants with UC were diagnosed at a later age (p = 0.003) and had a higher academic status (p = 0.045) than patients with CD (Table 1).

**Table 1:** Patient characteristics.

	Healthy control N = 166	Entire IBD cohort N = 505	Ulcerative colitis N = 156	Crohn’s disease N = 335
Female Sex; n (%)	109 (65.7)	441 (87.3)	132 (84.6)	298 (89%)
Age, median (range)	32 (18–69)	35 (15–70)	34.5 (15–63)	35.5 (16–67)
**Age at diagnosis**				
5–10 yrs; n (%)	NA	15 (3)	3 (1.9)	12 (3.6)
10–15 yrs; n (%)	NA	54 (10.7)	15 (9.6)	38 (11.3)
15–25 yrs; n (%)	NA	241 (47.7)	61 (41)	174 (51.9)
25–35 yrs; n (%)	NA	114 (22.6)	41 (26.3)	69 (20.6)
>35 yrs; n (%)		81 (16)	33 (21.2)	42 (12.5)
**Treating physician**				
Primary care; n (%)	NA	47 (93)	16 (10.3)	27 (8.1)
Specialist (GI); n (%)	NA	452 (89.9)	137 (87.8)	307 (91.6)
**Sick days per month**				
1-2d; n (%)		380 (75.2)	125 (80)	248 (74)
3-5d; n (%)		72 (14.3)	17 (10.9)	52 (15)
>5d; n (%)		53 (10.5)	14 (9)	35 (10.4)
Concommitant disease, n (%)	8 (4.8)	187 (37)	56 (35.9)	126 (37.6)
Recognized disability; n (%)	10 (6)	285 (56.4)	81 (51.9)	196 (58.5)
Recognized grade of disability %; median(range)	0 (0–100)	30 (0–100)	20 (0–100)	30 (0–100)
Overall emotional stress; median (range)	3.22 (1.18–5.0)	3.25 (1.04–5)	3.3 (1.11–4.96)	3.23 (1.04–5)
Overall discrimination at work; median (range)	3 (1–5)	4 (1–5)	4 (1–5)	4 (1–5)

**Table 1** shows the patients characteristics for all participants divided up into disease entities and control group.

A total of 56.4% (n = 285) in the entire IBD cohort and 51.9% (n = 81) in the UC and 58.5% (n = 196) CD subgroup had a recognized disability, whereas only 6% (n = 10) in the control group had a recognized disability (p<0.0001). No difference was observed between the IBD subgroups in terms of the numerical amount of recognized disabilities (p = 0.414). However, statistically significant differences were observed for the median disability scores between the IBD subgroups. Patients with CD had higher recognized disability scores (p = 0.034).

The majority of the patients saw a gastroenterologist rather than a primary care physician (452 vs. 47); however, no statistically significant difference was observed between the IBD groups (p = 0.807). Furthermore, no statistical difference between sick days between the IBD subgroups was observed (p = 0.162). For a detailed overview of participant characteristics, please see Table 1.

### Results of the online survey

**Overall stress and recognized disability:** Patients with IBD had a significantly increased level of everyday and work-related stress in the past six months in comparison to the healthy volunteers (p<0.001). Similar results were observed for stress levels in the past five years (p<0.001). Furthermore, statistically significant differences in the frequency of recognized disability (p<0.001) were observed between IBD and control group participants.

**Stress in daily life and work in IBD subjects:** No statistically significant differences were observed between UC and CD participants in terms of overall emotional stress in daily life in the past six months (p = 0.755) and five years (p = 0.239) as well as emotional stress at work in the past six months (p = 0.906) and past five years (p = 0.607). However, in the subsection investigating different aspects of daily live, significant differences concerning emotional stress could be observed. Results for participants in the UC group concerning leisure time and social interactions with friends in the past five years were significantly higher than in the CD group (p = 0.039), while no statistically significant results were observed for the same question related to the past 6 months (p = 0.997). Furthermore, in the subsection investigating different aspects of work live participants in the UC group experienced significantly more emotional stress than Crohn’s disease subjects (p = 0.030) concerning the fear of not fining a restroom in time in the past six months. Detailed information concerning the respective answers of this questionnaire segment is provided in (S1 and S2 Tables).

**Discrimination in daily life (i.e, private life) and work:** There was no statistically significant difference for overall discrimination between the IBD groups (p = 0.537). However, a tendency was observed that participants in the UC group reported higher values for emotional stress caused by negative comments of colleagues concerning their IBD (p = 0.057). Results are shown in detail in S3 Table.

**Contact with social care work and evaluation:** Across the IBD subgroups significantly more CD participants had been in contact with the social care system (p = 0.024), however, no statistically significant differences were observed concerning the other questions between the groups (S4 Table).

#### Factors independently associated with a recognized disability in IBD patients

Uni- and multivariate linear regression analyses were performed to explore associations with recognized disabilities in IBD participants. As shown in Table 2, academic status, sick days per month and fear of not reaching the restroom in time at work in the past six months and emotional IBD-related stress when meeting friends, were independently associated with a recognized disability. A low academic status was the strongest predictor for a recognized disability (multivariate p = 0.006; OR = 1.24 95% CI = 1.06–1.44, Table 2).

**Table 2:** Logistic regression analysis for parameters associated with recognized disability in IBD patients.

	Univariate analysis		Multivariate analysis	
	*p*-value	OR (95% CI)	p-value	OR (95% CI)
**IBD Cohort**				
Academic status	0.0001	1.33 (0.15–1.53)	0.006	1.24 (1.06–1.44)
Sick days/month	0.009	0.68 (0.51–0,90)	0.010	0.66 (0.48–0,90)
Work 6m: Emotional stress not reaching toilette in time	0.019	0.86 (0.77–0.97)		
Work 5y: Emotional stress not reaching toilette in time	0.0001	0.77 (0.68–0.87)	0.053	0.86 (0.74–1.00)
Private 5y: meeting friends	0.0001	0.75 (0.65–0.87)	0.013	0.80 (0.67–0.95)

**Table 2** shows the results of uni- and multivariate binary regression for parameters associated with recognized disability.

#### Factors independently associated with contact with social work in IBD patients

A recognized disability was the strongest predictor for having been in contact with social work (multivariate p<0.0001; OR = 4.17 95% CI = 2.62–6.64) together with the participant’s age (i.e., higher age) and CD. The number of sick days per month was statistically significant only in univariate analysis, and therefore obviously no independent predictor for contact with social work (Table 3).

**Table 3:** Logistic regression analysis for parameters associated with social work contact in IBD patients.

	Univariate analysis		Multivariate analysis	
	*p*-value	OR (95% CI)	p-value	OR (95% CI)
**IBD Cohort**				
**Sick days/month**	0.053	0.76 (0.58–1,03)		
**Recognized disability**	0.0001	2.79 (1.93–4.05)	0.0001	4.17 (2.62–6.64)
**Age**	0.0001	0.95 (0.94–0.97)	0.002	0.97 (0.95–0.99)
**Crohn’s disease**	0.003	0.55 (0.37–0.82)	0.013	0.59 (0.38–0.89)

**Table 3** shows the results of uni- and multivariate binary regression for parameters associated with social work contact.

## Discussion

IBD has a significant psychosocial impact on patients’ quality of life. Aside from physical disability, IBD patients experience more stress and discrimination in comparison to the general population[16]^,^[17] affecting not only emotional stress burden but productivity, academic and vocational training[18]^,^[19].

A large cohort study of the European Federation of Crohn’s and Ulcerative Colitis Associations revealed that over 48% of participants felt that IBD influenced their lives even in remission in comparison to non-IBD patients. Furthermore 56% of IBD patients in this study believed that their IBD affected their career path and led to psychological stress and disability[20]. Thus, over the last decade, concerted efforts have been made to understanding causes, determining predictors, and ultimately improving QoL and emotional stress in IBD patients[2]^,^[21]^,^[22].

Many physician-initiated studies and have focused on QoL in relation to clinical aspects such as disease activity, medical treatment, symptoms, and natural history of disease[2]^,^[23]^,^[24]. Of note, Lönnfors et al. have shown that the majority of participants in their study felt that they haven´t been asked all desired questions and wished their physician would have asked more probing questions[20]. Thus, certain issues are highly relevant to the patient and might be missed by the treating physician.

We, therefore, aimed to create a questionnaire focussing on issues of relevance for IBD patients. With particular focus on emotional stress, disability, and satisfaction with the social care system, importantly, including a control group to serve as a reference for the baseline characteristics such as age, disability, sick days, and education.

Interestingly participants with UC were diagnosed at a later age and had higher academic training than patients with CD. Possibly suggesting that patients with CD, as they were diagnosed younger in our cohort, experienced limitations in their education. A Swedish study showed that CD patients did indeed worry more about achievement and complications than about stigmatization and intimacy[18].

Furthermore, participants in the IBD group were significant more likely to have a recognized disability and more sick days per month, compared to the control group. The subgroup analysis revealed that participants with CD had statistically significant higher disability scores than patients with UC.

We identified a couple of parameters independently associated with having a recognized disability. A low academic status was the strongest predictor for having a recognized disability.

A Spanish study evaluating disability in IBD patients determined age, time to diagnosis, CD, perianal disease, active disease, incontinence, need for psychological or anti-TNF treatment, surgeries, and number of medical visits and tests in the past year as predictors for disability in their cohort[23]. Hence, some of the predictors determined in our study appear to be in line with the study mentioned above.

Participants with IBD reported significantly higher levels of everyday and work-related stress in the past six months as well as in the past five years in comparison to the control group. Participants in the UC group reported significantly higher levels of stress associated with social contacts and caused by the fear of not reaching a restroom in time. These results might be due to the fact that stool frequency is often higher in UC patients.

Another study has shown that the fear of not having a restroom in close proximity is of high relevance to IBD patients and results in reduced QoL[20]. A study by Rubin et al. showed that, compared to patients with other chronic illnesses, patients with UC worry significantly more often over embarrassment, depression, and disease complications[24].

Only 155 IBD participants (30.7%) had contact with the German social care system, with no differences observed between the IBD subgroups. Logistic regression analysis determined a recognized disability, a younger patient’s age, and CD to be independently associated with having been in contact with social work/care. Interestingly the majority of the patients saw a gastroenterologist rather than a primary care physician (GP; 452 vs. 47). However, whether patients were cared for by a specialist or by a GP was neither a predictor for a recognized disability nor for having been in contact with social work. While IBD patients benefit from being treated by a specialist rather than a GP[25], our study shows that this does not apply for having a recognized disability or having been in contact with the social support system or support groups.

It might be useful for physicians to promote self-help groups and social care, taking into account that it may be easier for IBD patients to talk to peers about certain disease-related topics. Van der Eijk and colleagues were able to show that the quality of care the patients received influenced their health-related quality of life (HRQoL)[26]. Furthermore, social factors have been identified as being relevant to treatment adherence[27], and self-mangement[28] in young IBD patients and that practical social support is associated with treatment adherence[29].

Limitations to our study are for one that we did not obtain disease-specific data, and it is, therefore, unclear if disease activity influences a patient’s response to the survey. The concomitant diseases reported by some of the patients (i.e., depression) could also influence the answers given in the questionnaire. Furthermore, it is possible that retrospective judgments are subject to cognitive recollection biases and could thus call the reliability of the data into question[30]. The majority of participants were female, possibly highlighting the willingness of female patients to be active on social media platforms, participate in questionnaires, and verbalize their emotions. However, it has been previously described that female patients do react differently[31], for example, to pain[32], and thus, gender biases in our cohort cannot be entirely discounted. One might also argue that with the abundance of evaluated IBD QoL evaluation tools, we should have applied one of these as a supplementary or as a control evaluation tool. However, as we specifically sought to evaluate social care, disability, and discrimination from a patient perspective, these needs were not entirely met by existing questionnaires. The presented study should be considered a pilot-study as we aimed to include patients with IBD and their point-of-views. We are currently working on a survey, generated with validated tools. Lastly, this questionnaire was in German, had to be completed online, and most participants learned about it through social media. Therefore possibly some patient cohorts were not included, such as those of advanced age, with limited German language skills or no online access.

In summary, our study provides real-world data in a large German IBD cohort on factors associated with emotional stress and disability. We were able to determine factors independently associated with disability and have been in contact with social work. Remarkably over 500 IBD patients participated in this study advertised on social media—demonstrating its impact on a younger demographic. Furthermore, only a fraction of the patients has been in contact with some form of social support, showing that these patients are probably underserved by social services. Health-care professionals should be aware of this deficiency and promote social work and contact with the social system to further improve QoL in IBD.

## Supporting information

S1 File Questionnaire. Questionnaire on emotional stress, discrimination, disability and contact/support by the social care system.
(DOCX)

S1 Table Emotional stress in daily life. Results of the subsections of the online survey focused on emotional stress in daily life.
(ODT)

S2 Table Emotional stress at work. Results of the subsections of the online survey focused on emotional stress at work.
(ODT)

S3 Table Discrimination in private life and at work. Results of the subsection of the online survey focused on discrimination in private life and at work.
(ODT)

S4 Table Social Work Contact and Evaluation. Results of the subsection of the online survey focused on social work and its evaluation.
(ODT)

## References

[1] Ghosh S, Mitchell R. Impact of inflammatory bowel disease on quality of life: Results of the European Federation of Crohn’s and Ulcerative Colitis Associations (EFCCA) patient survey. J Crohn’s Colitis. 2007;9;1(1):10–20. 21172179 10.1016/j.crohns.2007.06.005

[2] Muller KR, Prosser R, Bampton P, Mountifield R, Andrews JM. Female gender and surgery impair relationships, body image, and sexuality in inflammatory bowel disease: Patient perceptions. Inflamm Bowel Dis. 2010;4;16(4):657–63. 10.1002/ibd.21090 19714755

[3] Sellin J. Disability in IBD: The devil is in the details. Inflammatory Bowel Diseases. 2010;1;16(1):23–6. 10.1002/ibd.21014 19526528

[4] Spekhorst LM, Oldenburg B, Van Bodegraven AA, De Jong DJ, Imhann F, Van De Meulen-De Jong AE, et al Prevalence of-And risk factors for work disability in Dutch patients with inflammatory bowel disease. World J Gastroenterol. 2017;(12 14;23(46):8182–8192.). 10.3748/wjg.v23.i46.8182 29290654 PMC5739924

[5] Vester-Andersen MK, Prosberg M V., Vind I, Andersson M, Jess T, Bendtsen F. Low Risk of Unemployment, Sick Leave, and Work Disability Among Patients with Inflammatory Bowel Disease. Inflamm Bowel Dis. 2015;10;21(10):2296–303. 10.1097/MIB.0000000000000493 26164663

[6] MacKner LM, Bickmeier RM, Crandall W V. Academic achievement, attendance, and school-related quality of life in pediatric inflammatory bowel disease. J Dev Behav Pediatr. 2012;2;33(2):106–11. 10.1097/DBP.0b013e318240cf68 22267107

[7] Carreon SA, Bugno LT, Wojtowicz AA, Greenley RN. School Functioning in Adolescents with Inflammatory Bowel Diseases: An Examination of Disease and Demographic Correlates. Inflamm Bowel Dis. 2018;7 12;24(8):1624–1631. 10.1093/ibd/izy026 29718311

[8] Assa A, Ish-Tov A, Rinawi F, Shamir R. School attendance in children with functional abdominal pain and inflammatory bowel diseases. J Pediatr Gastroenterol Nutr. 2015;Nov;61(5):553–7. 10.1097/MPG.0000000000000850 25950089

[9] Taft TH, Keefer L. A systematic review of disease-related stigmatization in patients living with inflammatory bowel disease. Clinical and Experimental Gastroenterology. 2016 p. 3 7;9:49–58. 10.2147/CEG.S83533 27022294 PMC4789833

[10] Büsch K, da Silva SA, Holton M, Rabacow FM, Khalili H, Ludvigsson JF. Sick leave and disability pension in inflammatory bowel disease: A systematic review. Journal of Crohn’s and Colitis. 2014 p. 11;8(11):1362–77. 10.1016/j.crohns.2014.06.006 25001582

[11] Høivik ML, Moum B, Solberg IC, Henriksen M, Cvancarova M, Bernklev T. Work disability in inflammatory bowel disease patients 10 years after disease onset: Results from the IBSEN study. Gut. 2013;3;62(3):368–75. 10.1136/gutjnl-2012-302311 22717453

[12] Choi K1, Chun J2, 3, Han K4, Park S5, Soh H6, Kim J7, Lee J8, Lee HJ9, Im JP10, Kim JS11 12. Risk of Anxiety and Depression in Patients with Inflammatory Bowel Disease: A Nationwide, Population-Based Study. J Clin Med. 2019;5 10;8(5). 10.3390/jcm8050654 PMC6572298 31083476

[13] van den Brink G, Stapersma L, Vlug LE, Rizopolous D, Bodelier AG, van Wering H, et al Clinical disease activity is associated with anxiety and depressive symptoms in adolescents and young adults with inflammatory bowel disease. Aliment Pharmacol Ther. 2018;8;48(3):358–369. 10.1111/apt.14832 29897134

[14] Lewis K, Marrie RA, Bernstein CN, Graff LA, Patten SB, Sareen J, et al The Prevalence and Risk Factors of Undiagnosed Depression and Anxiety Disorders Among Patients With Inflammatory Bowel Disease. Inflamm Bowel Dis. 2019;19 pii: izz045 10.1093/ibd/izz045 [Epub ah. 30888037

[15] World Health Organization. International Classification of Functioning, Disability and Health (ICF). Geneva: WHO, 2001. http://www.who.int/classifications/icf/en (accessed 27 Dec 2010).

[16] Romberg-Camps MJL, Bol Y, Dagnelie PC, Hesselink-Van De Kruijs MAM, Kester ADM, Engels LGJB, et al Fatigue and health-related quality of life in inflammatory bowel disease: Results from a population-based study in the Netherlands: The IBD-South Limburg cohort. Inflamm Bowel Dis. 2010;12;16(12):2137–47. 10.1002/ibd.21285 20848468

[17] Haapamäki J, Roine RP, Sintonen H, Turunen U, Färkkilä MA, Arkkila PET. Health-related quality of life in inflammatory bowel disease measured with the generic 15D instrument. Qual Life Res. 2010;8;19(6):919–28. 10.1007/s11136-010-9650-4 20361263

[18] Stjernman H, Tysk C, Almer S, Ström M HH. Worries and concerns in a large unselected cohort of patients with Crohn’s disease. Scand J Gastroenterol. 2010;6;45(6):696–706. 10.3109/00365521003734141 20334474

[19] Freckmann M, Seipp A, Laass MW, Koletzko S, Claßen M, Ballauff A, et al School-related experience and performance with inflammatory bowel disease: Results from a cross-sectional survey in 675 children and their parents. BMJ Open Gastroenterol. 2018;11 24;5(1):e000236 10.1136/bmjgast-2018-000236 30538821 PMC6254744

[20] Lönnfors S, Vermeire S, Greco M, Hommes D, Bell C, Avedano L. IBD and health-related quality of life—Discovering the true impact. J Crohn’s Colitis. 2014;10;8(10):1281–6. 24662394 10.1016/j.crohns.2014.03.005

[21] Sainsbury A, Heatley R V. Review article: Psychosocial factors in the quality of life of patients with inflammatory bowel disease. Alimentary Pharmacology and Therapeutics. 2005 p. 3 1;21(5):499–508. 10.1111/j.1365-2036.2005.02380.x 15740531

[22] Irvine EJ. Quality of life of patients with ulcerative colitis: Past, present, and future. Inflammatory Bowel Diseases. 2008 p. 4;14(4):554–65. 10.1002/ibd.20301 17973299

[23] Ramos A, Calvet X, Sicilia B, Vergara M, Figuerola A, Motos J, et al IBD-related work disability in the community: Prevalence, severity and predictive factors. A cross-sectional study. United Eur Gastroenterol J. 2015;8;3(4):335–42. 10.1177/2050640615577532 PMC4528207 26279841

[24] Rubin DT, Dubinsky MC, Panaccione R, Siegel CA, Binion DG, Kane S V., et al The impact of ulcerative colitis on patients’ lives compared to other chronic diseases: A patient survey. Dig Dis Sci. 2010;4;55(4):1044–52. 10.1007/s10620-009-0953-7 20155319

[25] Louis E, Dotan I, Ghosh S, Mlynarsky L, Reenaers C, Schreiber S. Optimising the inflammatory bowel disease unit to improve quality of care: Expert recommendations. Journal of Crohn’s and Colitis. 2015 p. 8; 9(8): 685–691. 10.1093/ecco-jcc/jjv085 25987349 PMC4584566

[26] van der Eijk I, Vlachonikolis IG, Munkholm P, Nijman J, Bernklev T, Politi P, et al The role of quality of care in health-related quality of life in patients with IBD. Inflamm Bowel Dis. 2004;7;10(4):392–8. 10.1097/00054725-200407000-00010 15475747

[27] Hommel KA, Baldassano RN. Brief report: Barriers to treatment adherence in pediatric inflammatory bowel disease. J Pediatr Psychol. 2010;10;35(9):1005–10. 10.1093/jpepsy/jsp126 20026567 PMC2948828

[28] Plevinsky JM, Greenley RN, Fishman LN. Self-management in patients with inflammatory bowel disease: Strategies, outcomes, and integration into clinical care. Clinical and Experimental Gastroenterology. 2016 p. 8 23;9:259–67. 10.2147/CEG.S106302 27601930 PMC5003515

[29] DiMatteo MR. Social Support and Patient Adherence to Medical Treatment: A Meta-Analysis. Health Psychology. 2004 p. 3;23(2):207–18. 10.1037/0278-6133.23.2.207 15008666

[30] Althubaiti A. Information bias in health research: Definition, pitfalls, and adjustment methods. Journal of Multidisciplinary Healthcare. 2016 p. 5 4;9:211–7. 10.2147/JMDH.S104807 27217764 PMC4862344

[31] Helbig S, Backhaus J. “Sex differences in a real academic stressor, cognitive appraisal and the cortisol response.” Physiol Behav. 2017;10 1;179:67–74. 10.1016/j.physbeh.2017.05.027 28546084

[32] Belfer I. Pain in women. Agri. 2017 p. 4;29(2):51–54. 10.5505/agri.2017.87369 28895988

