# Supplementary material for: Real-world data about emotional stress, disability and need for social care in a German IBD patient cohort
Source: PLoS One. 2020 Jan 3;15(1):e0227309. doi: 10.1371/journal.pone.0227309 (PMC6941800; doi:10.1371/journal.pone.0227309)
Supplement: S1 File — Questionnaire on emotional stress, discrimination, disability and contact/support by the social care system. (DOCX) [file pone.0227309.s001.docx]

**Questionnaire on emotional stress, discrimination, disability and contact/support by the social care system**

*(All sections of the questionnaire were – in part or with modifications – taken into consideration for the non-IBD participants in our final evaluation)*

**Section 1:** General Information

| **CE01** Age**:** |
| --- |

|  |
| --- |
| **CE02** Gender  1 = Male 2 = Female 3 = Other |

| **CE03** Citizenship  1 = German 2 = Other |
| --- |

| **CE04** Faith  1 = Christian  2 = Jewish  3 = Mulim |
| --- |
| **CE05** Education  1 = Hauptschule (=9th grade diploma)  2 = Realschule (=10th grade diploma)  3 = High school diploma  4 = Technical baccalaureate  5 = University degree  6 = no graduation |

| **CE06** Employment status  1 = in employment  2 = college student  3 = student  4 = retired  5 = unable to work  6 = unemployed/looking for employment  7 = other |
| --- |

| **CF01** What IBD do you have?  1 = Ulvcerative colitis  2 = Crohn’s disease  3 = IBD unclassified |
| --- |
|  |

| **CF02** How old were you when IBD was diagnosed**?**  1 = 5-10 years 2 = 10-15 years 3 = 15-25 years  4 = 25-35 years 5 = >35 years |
| --- |
|  |

| **CF03** Who is treating you for your IBD?  1 = General practioner  2 = Specialist (Gastroenterologist, Proktologist)  3 = other  If other was chosen, please specify: _______________________ |
| --- |

| **CF04** Sick days  1 = 1-2 days  2 = 3-5 days  3 = >5 days |
| --- |

| **CF05** Specification number of recognized disability: |
| --- |

| **CF06** "Aside from your IBD, what other disease were taken into consideration when the grade of disability was evaluated? |
| --- |

| **CF07** Do you believe the grade of recognized disability was evaluated and granted justly?  1 = Yes  2 = No I believe it was evaluated as being too low  3 = No I believe it was evaluated as being too high |
| --- |

| **CF8** If you answered the previous question eith “no“, please state which grade would have been adeqaute in your opinion. |
| --- |

**Please answer the questions in the following sections with “yes“ or “no“ and along the provided scale [Scale (1-5; 1= not at all, 5= severe)] if indicated.**

**Section 2:** Emotional stress in daily life

| **CG01** Overall emotional stress? |
| --- |
|  |
| **Emotional stress in daily life over the past six months** |
| **CG01_01** How much does IBD affects your daily life/causes emotional stress in daily life?  **CG01_02** How much does IBD affects your daily life/causes emotional stress when doing household chores (for example cleaning, shopping)?  **CG01_03** How much does IBD affects your daily life/causes emotional stress in body hygiene?  **CG01_04** How much does IBD affects your daily life/causes emotional stress when being outside and fearing of not finding a restroom in time?  **CG01_05** How much does IBD affects your daily life/causes emotional stress in social activities (for example meeting friends)?  **CG01_06** How much does IBD affects your daily life/causes emotional stress when doing sports?  **CG01_07** How much does IBD affects your daily life/causes emotional stress when attending family activities/events?  **CG01_08** To what extent to you experience general exhaustion due to your IBD?  **CG01_09** How much does IBD affects your daily life/causes emotional stress when meeting/getting to know new people?  **CG01_10** How much does IBD affects your daily life/causes emotional stress in daily life when looking for /finding a new partner?  **CG01_11** How much does IBD affects your daily life/causes emotional stress in your relationship?  **CG01_12** How much does IBD affects your daily life/causes emotional stress in your sex life?  **CG01_13** How much does IBD affects your daily life/causes emotional stress in your self-care?  **CG01_14** How much does IBD affects your daily life/causes emotional stress in planning a vacation? |
| **Emotional stress in daily life over the past five years** |
| **CG02_01** How much does IBD affects your daily life/causes emotional stress in daily life?  **CG02_02** How much does IBD affects your daily life/causes emotional stress when doing household chores (for example cleaning, shopping)?  **CG02_03** How much does IBD affects your daily life/causes emotional stress in body hygiene?  **CG02_04** How much does IBD affects your daily life/causes emotional stress when being outside and fearing of not finding a restroom in time?  **CG02_05** How much does IBD affects your daily life/causes emotional stress in social activities (for example meeting friends)?  **CG02_06** How much does IBD affects your daily life/causes emotional stress when doing sports?  **CG02_07** How much does IBD affects your daily life/causes emotional stress when attending family activities/events?  **CG02_08** To what extent to you experience general exhaustion due to your IBD?  **CG02_09** How much does IBD affects your daily life/causes emotional stress when meeting/getting to know new people?  **CG02_10** How much does IBD affects your daily life/causes emotional stress in daily life when looking for /finding a new partner?  **CG02_11** How much does IBD affects your daily life/causes emotional stress in your relationship?  **CG02_12** How much does IBD affects your daily life/causes emotional stress in your sex life?  **CG02_13** How much does IBD affects your daily life/causes emotional stress in your self-care?  **CG02_14** How much does IBD affects your daily life/causes emotional stress in planning a vacation? |

**Section 3:** Emotional stress in work life

| **Emotional stress in work life over the past six months** |
| --- |
| **CG03_01** How much does IBD affects your daily life/causes emotional stress in your work life?  **CG03_02** How much does IBD affects your daily life/causes emotional stress on your way to work?  **CG03_03** How much does IBD affects your daily life/causes emotional stress when fearing to not find a restroom in time?  **CG03_04** How much does IBD affects your daily life/causes emotional stress when working with your colleagues?  **CG03_05** How much does IBD affects your daily life/causes emotional stress in your work flow?  **CG03_06** How much does IBD affects your daily life/causes emotional stress when coordinating your work schedule?  **CG03_07** How much does IBD affects your daily life/causes emotional stress when a unexpected sitaution arises (for example a higher work load, filling in for a colleague)? |

| **Emotional stress in work life over the past six months** |
| --- |
| **CG04_01** How much does IBD affects your daily life/causes emotional stress in your work life?  **CG04_02** How much does IBD affects your daily life/causes emotional stress on your way to work?  **CG04_03** How much does IBD affects your daily life/causes emotional stress when fearing to not find a restroom in time?  **CG04_04** How much does IBD affects your daily life/causes emotional stress when working with your colleagues?  **CG04_05** How much does IBD affects your daily life/causes emotional stress in your work flow?  **CG04_06** How much does IBD affects your daily life/causes emotional stress when coordinating your work schedule?  **CG04_07** How much does IBD affects your daily life/causes emotional stress when a unexpected sitaution arises (for example a higher work load, filling in for a colleague)? |

**Section 4:** Discrimination in daily/private life

| **Discrimination in private life** |
| --- |
| **CH01**  Overall discrimination in the past five years (i.e. have you experienced discrimination) and how much has this affected you (scale1-5)  1 = yes  2 = no |

| **CH02** Have you experienced discrimination by your family and how much has this affected you (scale1-5)  Examples: “Others are worse off then you?“, “You are complaing so much!“  1 = yes  2 = no |
| --- |

| **CH03 Have you ever had to lie about or concerning something about your disease and how much has this affected you (scale1-5)**  **1 = yes**  **2 = no** |
| --- |

| **CH04** Have you ever experienced discrimination in a restaurant or shop? For example: you were not permitted to use their restroom and how much has this affected you (scale1-5)  1 = yes  2 = no |
| --- |

| **CH05** Have you ever had to lie about the stress caused by your disease or your health state to other people and how much has this affected you (scale1-5)  1 = yes  2 = no |
| --- |

| **Discrimination at work** |
| --- |
| **CI01** Overall discrimination at work (i.e. have you experienced discrimination) and how much has this affected you (scale1-5)  1 = yes  2 = no |

| **CI02** Did you ever go to work, even though you were sick and how much has this affected you (scale1-5)  1 = yes  2 = no |
| --- |
|  |
| **CI03** Have you ever had to hear negative remarks concerning your disease from your superior/boss and how much has this affected you (scale1-5)  1 = yes  2 = no |

| **CI04** Did you ever got to work earlier due to your disease and how much has this affected you (scale1-5)  1 = yes  2 = no |
| --- |

| **CH05** Have you ever experienced that harder work was assigned to you after a sick leave and how much has this affected you (scale1-5)  1 = yes  2 = no |
| --- |

| **CH06** Have you ever experienced that lighter/easier work was assigned to you after a sick leave and how much has this affected you (scale1-5)  1 = yes  2 = no |
| --- |

| **CH07** Have you ever experienced/heard negative/jealous remarks from your colleagues concerning your disease and how much has this affected you (scale1-5)  For example: „“I am jealous that you get xxxx“  1 = yes  2 = no |
| --- |

**Section 5:** Social care system

| **CK04_01** Have you ever been in contact with the social care system?  For example: during hospital stay, rehabilitation  1 = yes  2 = no |
| --- |

**If you answered the previous question with “yes“ what was your experience concerning the following aspects.**

**Please use a scale from 1-5 if indicared. 1= highly dissatisfied, 5= very satisfied**

| **CK04_02** Overall satiscaftion with social worker contact?  **CK04_03** Consulation (were questions answered or infromation provided to your satisfaction)?  **CK04_04** Reachability (was it easy to contact the respecitve party)?  **CK04_05** Comprehensibility (were even complex or diffcult information explained to you adequately?  **CK04_06** Providing information concerning IBD services?  **CK04_07** Providing information concerning IBD groups/organisations?  **CK04_08** Providing information concerning rehabilitation?  **CK04_09**. Providing information concerning care levels, early retirement?  **CK04_10**. Providing information and helping to find care services?  **CK04_11**. Providing information and helping to find stoma care?  **CK04_12.** Providing information and helping to find a physician/expert? |
| --- |
