# Supplementary material for: Real-world data about emotional stress, disability and need for social care in a German IBD patient cohort
Source: PLoS One. 2020 Jan 3;15(1):e0227309. doi: 10.1371/journal.pone.0227309 (PMC6941800; doi:10.1371/journal.pone.0227309)
Supplement: Supplementary file 2 [file pone.0227309.s002.odt]

S1 Table. Emotional stress in daily life

|  | Entire IBD cohort N=505 | Ulcerative colitis N=156 | Crohn’s disease N=335 | p-Value |
| --- | --- | --- | --- | --- |
| Emotional stress: Daily life 6 months, mean (SD) |  |  |  |  |
| 1. Stress daily life | 3,41 (1.027) | 3.42 (1.060) | 3.42 (1.003) | 0.719 |
| 2. Everyday household chores | 2.84 (1.198) | 2.74 (1.095) | 2.89  (1.241) | 0.228 |
| 3. Body hygiene | 2.02 (1.154) | 1.95 (1.157) | 2.05 (1.143) | 0.252 |
| 4. Not finding a toilet in time | 3.43 (1.346) | 3.54 (1.346) | 3.38 (1.336) | 0.180 |
| 5. Social activities | 3.28 (1.209) | 3.28 (1.200) | 3.28 (1.200) | 0.997 |
| 6. Sports | 3.48 (1.231) | 3.46 (1.220) | 3.50 (1.230) | 0.730 |
| 7. Family activities/events | 2.84 (1.192) | 2.78 (1.171) | 2.87 (1.197) | 0.450 |
| 8. General exhaustion | 3.86 (1.062) | 3.87 (1.066) | 3.87 (1.056) | 0.951 |
| 9. Meeting new people | 2.94 (1.354) | 3.02 (1.407) | 2.91 (1.324) | 0.361 |
| 10. Finding a new partner | 2.90 (1.416) | 2.95 (1.463) | 2.90 (1.387) | 0.739 |
| 11. Romantic relationship | 2.81 (1.265) | 2.85 (1.335) | 2.79 (1.231) | 0.707 |
| 12. Sex life | 3.11 (1.290) | 3.10 (1.309) | 3.13 (1.275) | 0.830 |
| 13. Self-care | 2.95 (1.205) | 2.94 (1.184) | 2.97 (1.205) | 0.927 |
| 14. Planning a vacation | 3.46 (1.295) | 3.15 (1.250) | 3.43 (1.275) | 0.260 |
| Emotional stress: Daily life 5 years, mean (SD) |  |  |  |  |
| 1. Stress daily life | 3.68 (1.034) | 3.76 (1.036) | 3.64 (1.033) | 0.222 |
| 2. Everyday household chores | 3.66 (1.039) | 3.68 (1.065) | 3.65 (1.029) | 0.653 |
| 3. Body hygiene | 3.11 (1.247) | 3.13 (1.241) | 3.10 (1.241) | 0.847 |
| 4. Not finding a toilet in time | 2.45 (1.335) | 2.41 (1.334) | 2.47 (1.323) | 0.625 |
| 5. Social activities | 3.66 (1.254) | 3.82 (1.208) | 3.57 (1.265) | 0.039 |
| 6. Sports | 3.50 (1.180) | 3.57 (1.166) | 3.45 (1.183) | 0.317 |
| 7. Family activities/events | 3.65 (1.177) | 3.64 (1.154) | 3.67 (1.177) | 0.711 |
| 8. General exhaustion | 3.11 (1.275) | 3.14 (1.277) | 3.09 (1.273) | 0.687 |
| 9. Meeting new people | 3.89  (1.116) | 3.88 (1.085) | 3.90 (1.118) | 0.712 |
| 10. Finding a new partner | 3.01 (1.338) | 3.13 (1.326) | 2.96 (1.333) | 0.175 |
| 11. Romantic relationship | 2.98 (1.358) | 3.11 (1.367) | 2.93 (1.334) | 0.164 |
| 12. Sex life | 3.03 (1.286) | 3.13 (1.232) | 2.98 (1.300) | 0.236 |
| 13. Self-care | 3.20 (1.264) | 3.25 (1.272) | 3.19 (1.255) | 0.627 |
| 14. Planning a vacation | 3.18 (1.258) | 3.22 (1.267) | 3.15 (1.250) | 0.542 |

S1 Table Results of the subsections of the online survey focused on emotional stress in daily life.
