# Supplementary material for: Real-world data about emotional stress, disability and need for social care in a German IBD patient cohort
Source: PLoS One. 2020 Jan 3;15(1):e0227309. doi: 10.1371/journal.pone.0227309 (PMC6941800; doi:10.1371/journal.pone.0227309)
Supplement: Supplementary file 3 [file pone.0227309.s003.odt]

S2 Table. Emotional stress at work

|  | Entire IBD cohort N=505 | Ulcerative colitis N=156 | Crohn’s disease N=335 | p-Value |
| --- | --- | --- | --- | --- |
| Stress at work: 6 months, mean (SD) |  |  |  |  |
| 1. General stress at work | 3.43 (1.365) | 3.58 (1.312) | 3.36 (1.381) | 0.102 |
| 2. Way to work | 3.25 (1.333) | 3.31 (1.301) | 3.23 (1.338) | 0.556 |
| 3. Not finding a toilet in time | 2.79 (1.505) | 3.02 (1.480) | 2.69 (1.508) | 0.030 |
| 4. Working with colleagues | 3.46 (1.556) | 3.47 (1.487) | 3.45 (1.580) | 0.914 |
| 5. Workflow | 2.51 (1.319) | 2.65 (1.240) | 2.44 (1.339) | 0.106 |
| 6. Coordinating work schedule | 2.60 (1.311) | 2.69 (1.291) | 2.54 (1.298) | 0.293 |
| 7. Spontaneous situations | 2.65 (1.388) | 2.71 (1.278) | 2.62 (1.427) | 0.562 |
| Stress at work: 5 years, mean (SD) |  |  |  |  |
| 1. General stress at work | 3.24 (1.366) | 3.31 (1.323) | 3.22 (1.376) | 0.551 |
| 2. Way to work | 3.44 (1.343) | 3.53 (1.260) | 3.44 (1.354) | 0.544 |
| 3. Not finding a toilet in time | 3.01 (1.481) | 3.26 (1.426) | 3.00 (1.489) | 0.078 |
| 4. Working with colleagues | 3.52 (1.487) | 3.64 (1.439) | 3.46 (1.502) | 0.252 |
| 5. Workflow | 2.72 (1.373) | 2.81 (1.364) | 2.70 (1.362) | 0.463 |
| 6. Coordinating work schedule | 2.82 (1.347) | 2.89 (1.316) | 2.81 (1.347) | 0.580 |
| 7. Spontaneous situations | 2.91 (1.401) | 2.98 (1.378) | 2.90 (1.397) | 0.594 |

S2 Table Results of the subsections of the online survey focused on emotional stress at work.
