# Supplementary material for: Real-world data about emotional stress, disability and need for social care in a German IBD patient cohort
Source: PLoS One. 2020 Jan 3;15(1):e0227309. doi: 10.1371/journal.pone.0227309 (PMC6941800; doi:10.1371/journal.pone.0227309)
Supplement: Supplementary file 4 [file pone.0227309.s004.odt]

S3 Table. Discrimination in private life and at work

|  | Entire IBD cohort N=505 | Ulcerative colitis N=156 | Crohn’s disease N=335 | p-Value |
| --- | --- | --- | --- | --- |
| 1. Discrimination in private life, mean (SD) | 4.52 (0.803) | 1.59 (0.493) | 4.56 (0.702) | 0.833 |
| 2. Discrimination in the family | 4.46 (0.847) | 4.49 (0.739) | 4.45 (0.887) | 0.847 |
| 3. Hide the disease | 3.99 (1.104) | 4.07 (1.024) | 3.99 (1.113) | 0.638 |
| 4. Discrimination at a restaurant | 4.82 (0.509) | 4.84 (0.528) | 4.81 (0.504) | 0.317 |
| 5. Lied concerning disease-related stress | 1.21 (0.406) | 3.94 (1.080) | 3.88 (1.094) | 0.599 |
| 1. Discrimination at work 5 years, mean (SD) | 4.42 (0.912) | 4.47 (0.924) | 4.41 (0.874) | 0.509 |
| 2. Going to work sick | 4.13 (0.897) | 4.21 (0.858) | 4.10 (0.907) | 0.260 |
| 3. Remarks from Boss | 4.67 (0.640) | 4.74 (0.642) | 4.63 (0.646) | 0.057 |
| 4. Going to work earlier | 3.44 (1.381) | 3.54 (1.340) | 3.39 (1.403) | 0.326 |
| 5. Hard physical or mental work | 4.58 (0.733) | 4.4 (0.847) | 4.66 (0.655) | 0.104 |
| 6. Light/easy physical or mental work | 2.62 (1.221) | 2.32 (1.107) | 2.72 (1.250) | 0.133 |
| 7. Negative/Jealous remarks at work | 4.41 (0.899) | 4.38  (0.968) | 4.42 (0.881) | 0.902 |

S3 Table. Results of the subsection of the online survey focused on discrimination in private life and at work.
