# Supplementary material for: Real-world data about emotional stress, disability and need for social care in a German IBD patient cohort
Source: PLoS One. 2020 Jan 3;15(1):e0227309. doi: 10.1371/journal.pone.0227309 (PMC6941800; doi:10.1371/journal.pone.0227309)
Supplement: Supplementary file 5 [file pone.0227309.s005.odt]

S4 Table. Social Work Contact and Evaluation

|  | Entire IBD cohort N=505 | Ulcerative colitis N=156 | Crohn’s disease N=335 | p-Value |
| --- | --- | --- | --- | --- |
| Contact with social workers/support? |  |  |  |  |
| 1.Contact with social work N (yes) | 155 | 36 | 111 | 0.024 |
| 2.Overall satisfaction with social worker contact, mean (SD) | 3.85 (1.100) | 3.91 (0.914) | 3.83 (1.137) | 0.967 |
| 3. Consultation, mean (SD) | 148, 3.34 (1.221) | 3.38 (1.231) | 107, 3.30 (1.215) | 0.776 |
| 4. Reachability, mean (SD) | 136, 3.25 (1.216) | 27, 3.45 (0.985) | 100, 3.16 (1.261) | 0.286 |
| 5. Comprehensibility, mean (SD) | 138, 3.45 (1.202) | 32, 3.84 (1.019) | 99, 3.46 (1.240) | 0.158 |
| 6. Information concerning IBD services, mean (SD) | 113, 2.84 (1.379) | 25, 2.96 (1.274) | 82, 2.74 (1.413) | 0.479 |
| 7. Information concerning IBD groups/organisations | 118, 2.97 (1.411) | 27, 3.15 (1.433) | 86, 2.92 (1.408) | 0.477 |
| 8. Information concerning rehabilitation | 113, 3.14 (1.426) | 27, 3.04 (1.306) | 81, 3.12 (1.486) | 0.778 |
| 9. Information concerning care level, (early) retirement | 92, 2.75 (1.396) | 20, 2.95 (1.317) | 67, 2.67 (1.429) | 0.392 |
| 10. Information concerning care services | 48, 3.10 (1.356) | 14, 3.36 (1.336) | 32, 3.00 (1.391) | 0.426 |
| 11. Information concerning stoma care | 52, 3.37 (1.358) | 14, 3.07 (1.141) | 34, 3.41 (1.373) | 0.396 |
| 12. Information concerning physicians/experts, mean (SD) | 75, 2.68 (1.358) | 15, 2.47 (0.640) | 56, 2.68 (1.491) | 0.948 |

S4 Table. Results of the subsection of the online survey focused on social work and its evaluation.
